# Positive mental health in Slovenia before and during the COVID-19 pandemic

**DOI:** 10.3389/fpubh.2022.963545

**Published:** 2022-10-14

**Authors:** Matej Vinko, Petra Mikolič, Saška Roškar, Helena Jeriček Klanšček

**Affiliations:** National Institute of Public Health, Centre for Analysis and Development of Health, Ljubljana, Slovenia

**Keywords:** positive mental health, flourishing, languishing, COVID-19 pandemic, MHC-SF, resilience

## Abstract

**Background:**

Mental health has been heavily affected during the COVID-19 pandemic. In this study we compared the prevalence of flourishing and languishing mental health during the pandemic and examined which factors are associated with either category of positive mental health respectively.

**Methods:**

Data from two cross-sectional surveys with nationally representative samples of adult population in Slovenia conducted in 2019 (*n* = 9,047) and in 2021 (*n* = 3,429) are used. Positive mental health was measured with Mental Health Continuum–Short Form instrument. Logistic regression was used to examine the associations between flourishing and languishing mental health and relevant COVID-19 specific and other health-related factors.

**Results:**

There was a substantial decrease in the prevalence of flourishing and an increase in the prevalence of languishing mental health during the pandemic. Distribution of both flourishing and languishing mental health followed the socio-economic gradient. Resilience, COVID-19 literacy and changes in family relations, social interactions, and dietary habits were associated with both flourishing and languishing mental health.

**Conclusion:**

Positive mental health of the population worsened during the pandemic, more so in traditionally disadvantaged populations. Public health efforts need to be focused appropriately with an increased emphasis on strengthening resilience and health literacy.

## Introduction

The COVID-19 pandemic and policies enacted in response to the pandemic have greatly affected our lives. The prevalence of mental health issues has increased significantly during the pandemic, with certain population subgroups such as young adults, and individuals with low income and education suffering disproportionately, regardless of the severity of the pandemic or the type and scope of the government or public authorities' response to the pandemic (1–3). Mental health has long been defined merely as the absence of mental disorders. In recent years, the concept of mental health has changed and there is a strong consensus that mental health encompasses more than just the absence of psychopathology. Today, mental health is conceptualized in dual-continuum model, where mental health and mental disorders are related but distinct dimensions. Keyes referred to mental health as a “syndrome of symptoms of positive feelings and positive functioning in life” (4). In current literature mental health as defined by Keyes is often described as positive mental health (5). The presence of (positive) mental health is characterized as flourishing in life. Flourishing individuals are filled with positive emotion and are functioning well-psychologically and socially. Individuals who have low levels of (positive) mental health are described as languishing in life. Adults who are experiencing neither flourishing nor languishing in life are moderately mentally healthy. In this study we will use mental health when referring to the dual-continuum, encompassing both continuums or the complete mental health (both mental health and mental disorders continuums), and positive mental health (PMH) when referring to what Keyes defined as the mental health continuum.

A number of studies on PMH were conducted during the pandemic. Majority of them were of cross-sectional or repeated cross-sectional design, where changes in PMH throughout the pandemic were analyzed, but only few compared the levels of PMH before and during the pandemic (6–12). In our study we use two population-based surveys to compare levels of flourishing and languishing mental health in Slovenian adult population before and during COVID-19 pandemic and examine the association between sociodemographic, health-related factors and factors related to COVID-19 pandemic. Specifically, we aim to identify which changes in health-related lifestyle caused by the pandemic are associated with experiencing flourishing or languishing mental health.

## Methods

### Study population and period

We used nationally representative data from two population-based surveys of Slovenian adults. During COVID-19 pandemic, data were derived from the Survey on the impact of the pandemic on life (SI-PANDA), conducted from January 2021 to March 2021. Pre-pandemic estimates were derived from the European Health Interview Survey (EHIS), conducted in the spring and autumn of 2019.

#### COVID-19 pandemic sample

SI-PANDA is a research project on behavior insights on COVID-19 and pandemic fatigue in Slovenia. The research is conducted among the population aged 18 and over and is based on the World Health Organization tool for behavioral insights on COVID-19, adapted to the Slovenian context (13). The SI-PANDA survey was based on a two-stage sampling frame, stratified explicitly by size and type of settlement and implicitly by statistical region. All selected persons received a notification letter to access the online survey. Non-responders received paper questionnaires. There were 3830 participants (48.9 % response rate) in the SI-PANDA sample, 37.4% responded online and 62.6% in paper form. SI-PANDA includes questions and scales on socio-demographic characteristics, general health status, COVID-19 related health status, COVID-19 literacy, attitudes toward COVID-19 measures and vaccination, health related behavior, health care utilization, violence, and mental health.

#### Pre-pandemic sample

EHIS is a survey on health-related issues, run every 5 years. It is conducted on a sample of the Slovenian population aged 15 and over living in private households. Sampling was similar to the SI-PANDA survey, with a two stage sampling frame. There were 9,900 participants (67.2 % response rate) in the EHIS 2019 sample, 49.2% responded online and 50.8% in person (paper form). EHIS includes questions and scales on socio-demographic characteristics, health status, health related behavior, and health care utilization. In 2019 Slovenian EHIS questionnaire, a scale on PMH [Mental Health Continuum–Short Form (MHC-SF)] was also included.

### Measures

#### Outcome measure

PMH was the primary outcome measure. We used Mental Health Continuum – Short Form (MHC-SF), which measures PMH during last 30 days using 14 items on a 6-point scales from 0 (“never”) to 5 (“everyday”). The overall score reflects emotional, psychological and social well-being. Based on Keyes, mental health category (flourishing, moderate, and languishing) was computed for each participant (4). Flourishing mental health was indicated when a person felt at least one of the three emotional well-being symptoms and at least six of the eleven psychological and social well-being symptoms “every day” or “almost every day” in the past month. Languishing mental health was considered when participants reported they “never” or “one or twice” experienced at least one of the three emotional well-being symptoms and at least six of the eleven psychological and social well-being symptoms in the past month.

#### Socio-demographic measures

Socio-demographic data included information on sex, age, education level, employment status and marital status. All socio-demographic measures were defined as categorical variables. Sex included two categories: men and women. Age was defined with 7 groups: 18 to 24 years, 25 to 34 years, 35 to 44 years, 45 to 54 years, 55 to 64 years, 65 to 74 years, and 75 years or older. Education was classified into three groups: primary education or lower, secondary education and college education or higher. Employment status included 5 groups: employed or self-employed, student, retired, unemployed and other. Marital status was defined into 5 groups: single, married, living with a partner, widowed, and divorced.

#### Health and health-related behavior measures

The general health variables included the presence of at least one chronic health condition and the presence of at least one mental health disorder that were diagnosed more than 12 months ago. Participants assessed the impact of the pandemic on physical activity, healthy diet, sleep, and health status. Changes in drinking habits in last 12 months were also reported. Additionally, participants assessed the impact of the pandemic on family relations, financial security and social interaction with the extended family and friends, and reported whether they have experienced any kind of domestic violence (physical violence, psychological violence, sexual violence, economic violence or contact and freedom restriction) in past 12 months. A dichotomous variable was created on experiencing any type of domestic violence.

Participants reported whether they have been infected with COVID-19. COVID-19 health literacy questionnaire included nine items on a 7-point scale from 1 (“very difficult”) to 7 (“very easy”) to assess the ease/difficulty in finding information on symptoms and what to do if infected, understanding what authorities say, judging the reliability of the information, following recommendations and deciding on prevention behaviors. The questionnaire was adapted by WHO from Sørensen et al. and Griebler & Nitsche (13). We described the questionnaire by a two-factor model, using PCA with varimax rotation. We excluded item 4 due to low loadings (0.50) on both factors. The two-factor model accounted for 63% of the variance. Factor 1 described difficulty in understanding and following recommendations, while Factor 2 described difficulty in finding, understanding, and evaluating the information on coronavirus. The Score on Factor 1 was computed as an average score of items 5–9 and the score on Factor 2 was computed as the average score of items 1–3, with higher scores indicating a higher level of COVID-19 health literacy. Both scores showed adequate reliability, with Cronbach's alpha 0.86 (Factor 1) and 0.63 (Factor 2). Measure of resilience during the COVID-19 pandemic included three validated items on a 7-point scales from 1 (“strongly disagree”) to 7 (“strongly agree”), adapted by WHO from the brief resilience scale, indicating perceptions related to coping with stress and recovering (14). Items 1 and 3 were scored in reverse order so that a higher score on each item indicated higher coping ability. We described the resilience scale as a one-factor model by principal components analyses [PCA item 2 was excluded due to low loading (0.31)]. The resilience score was calculated as the average value of items 1 and 3. The model accounted for 82% of the variance and Cronbach's alpha showed adequate reliability (0.78).

### Statistical analysis

Data analyses were conducted in five steps. First, we calculated the socio-demographic characteristics of EHIS and SI-PANDA samples. Population weights on gender, age groups and statistical regions were used separately for both samples to achieve representativeness of the general population of Slovenia. Second, we estimated the distribution of PMH across categories (flourishing, moderate, languishing) before and during COVID-19 and calculated the difference and ratio of prevalence between the two samples. Third, we assessed the association between socio-demographic characteristics and prevalence of flourishing and languishing mental health in EHIS and SI-PANDA samples, using bivariable χ^2^ analysis. Fourth, we tested potential correlations between the same set of two binary outcomes and other independent variables in SI-PANDA sample. Fifth, we used multivariate logistic regression with flourishing and languishing mental health as dependent variables. In the first model (Adjusted model 1) we included baseline participants' characteristics as the independent variables and all measured variables in the second model (Adjusted model 2, forest plots for both outcomes are provided in Appendix 2). Before including independent variables in the model, we checked for multicollinearity between the non-categorical variables using variance inflation factor (VIF). The VIF values for all variables were <1.5, indicating low collinearity between them. Data analysis was carried out using the IBM Statistical Package for the Social Sciences (SPSS) version 25.

## Results

Final sample for 2019 EHIS study was 9,047 participants (we excluded 335 younger than 18 years and 518 with missing data on MHC-SF) and the final sample for 2021 SI-PANDA study was 3,429 participants (we excluded 401 participants with missing data on MHC-SF). Sociodemographic characteristics of study participants are presented in Appendix 1.

### Positive mental health before and during COVID-19 pandemic

In 2019, 61.5 % of adults in Slovenia had flourishing, 4.5 % had languishing mental health, and 34.1 % had moderate mental health. In 2021, the share of adults with flourishing mental health decreased significantly (38.6 %, Table 1), while the share of adults with languishing mental health increased almost twofold (8.0 %, Table 2). In both flourishing and languishing mental health, younger adults experienced largest deteriorations. While we see similar decreases in shares of flourishing adults for both males and females, there is a disproportionately larger increase in share of languishing women. Adults with low education had smallest share of flourishing individuals in both years and the same observation, albeit in opposite direction, holds true for languishing mental health. However, differences across educational strata are relatively small and do not reach the level of statistical significance. Comparison by employment status shows retired adults experienced least changes in PMH distribution, having biggest share of flourishing and smallest share of languishing individuals during the pandemic. Students and unemployed, on the other side, were most heavily affected. Comparison by marital status shows married and those living in extramarital union were most often flourishing and least often languishing in life both before and during COVID-19 pandemic.

**Table 1:** Comparison of prevalence of flourishing mental health before and during COVID-19 pandemic.

	**EHIS 2019**		**SI-PANDA 2021**	
	**% (95% CI)**	***p***	**% (95% CI)**	***p***
**Total**				
	61.5 (60.5–62.5)		38.6 (37.0–40.2)	
**Sex**		0.014		0.020
Female	60.2 (58.8–61.2)		36.6 (34.3–38.9)	
Male	62.7 (61.3–64.2)		40.5 (38.2–42.8)	
**Age (years)**		<0.001		<0.001
18–24	58.0 (54.5–61.5)		30.3 (25.4–35.7)	
25–34	61.0 (58.3–63.5)		32.1 (28.4–36.2)	
35–44	62.1 (59.8–64.4)		41.8 (38.1–45.5)	
45–54	59.3 (56.9–61.7)		36.5 (32.9–40.3)	
55–64	64.1 (61.7–66.4)		43.3 (39.4–47.3)	
65–74	65.9 (63.1–68.5)		42.5 (37.9–47.3)	
75 and more	57.6 (54.3–60.7)		40.6 (35.3–46.2)	
**Education status**		<0.001		0.051
primary education or lower	58.2 (55.9–60.5)		34.3 (30.6–38.1)	
secondary education	63.5 (62.1–64.8)		39.4 (37.2–41.6)	
college or higher	59.7 (57.7–61.7)		39.8 (36.7–42.9)	
**Employment status**		<0.001		<0.001
Employed, self-employed	63.3 (61.9–64.6)		38.1 (36.0–40.3)	
Student	54.0 (49.9–58.2)		28.5 (23.6–34.0)	
Retired	62.9 (61.1–64.7)		44.6 (41.4–47.8)	
Unemployed	52.1 (48.0–56.1)		29.8 (24.2–36.2)	
**Marital status**		<0.001		<0.001
Married	65.5 (64.1–66.9)		44.3 (41.8–46.8)	
Civil partner	59.7 (57.0–62.3)		39.0 (35.8–42.3)	
Single	55.6 (53.4–57.8)		27.1 (24.0–30.5)	
Widowed	57.6 (54.1–61.0)		36.2 (30.1–42.8)	
Divorced	56.6 (52.1–60.9)		32.5 (24.5–41.8)	

**Table 2:** Comparison of prevalence of languishing mental health before and during COVID-19 pandemic.

	**EHIS 2019**		**SI-PANDA 2021**	
	**% (95% CI)**	***p***	**% (95% CI)**	***p***
**Total**				
	4.5 (4.1–4.9)		8.0 (7.1–8.9)	
**Sex**		0.185		0.002
Female	4.7 (4.2–5.4)		9.4 (8.2–10.9)	
Male	4.2 (3.6–4.8)		6.6 (5.5–7.8)	
**Age (years)**		0.002		<0.001
18–24	5.7 (4.3–7.6)		14.9 (11.3–19.3)	
25–34	3.6 (2.7–4.7)		12.9 (10.3–15.9)	
35–44	4.2 (3.4–5.3)		6.9 (5.2–9.1)	
45–54	4.4 (3.5–5.5)		5.3 (3.8–7.4)	
55–64	4.1 (3.3–5.2)		5.2 (3.7–7.3)	
65–74	3.6 (2.7–4.8)		5.8 (3.9–8.4)	
75 and more	6.9 (5.5–8.8)		8.7 (6.1–12.4)	
**Education status**		<0.001		0.200
Primary education or lower	6.5 (5.5–7.8)		9.7 (7.6–12.2)	
Secondary education	4.0 (3.5–4.6)		7.8 (6.7–9.1)	
College or higher	3.9 (3.2–4.8)		7.2 (5.7–9.0)	
**Employment status**		<0.001		<0.001
Employed, self–employed	3.2 (2.8–3.8)		6.3 (5.3–7.5)	
Student	7.6 (5.6–10.1)		15.6 (11.9–20.3)	
Retired	4.4 (3.7–5.3)		6.1 (4.7–7.8)	
Unemployed	9.4 (7.3–12.1)		18.8 (14.3–24.5)	
**Marital status**		<0.001		<0.001
Married	3.5 (3.0–4.1)		4.6 (3.6–5.7)	
Civil partner	3.4 (2.5–4.5)		7.6 (6.0–9.5)	
Single	6.3 (5.2–7.4)		14.9 (12.5–17.7)	
Widowed	7.0 (5.4–9.0)		10.2 (6.8–15.0)	
Divorced	4.9 (3.3–7.2)		11.5 (6.8–18.8)	

### Flourishing mental health during COVID-19

Shares of individuals with flourishing mental health (Table 3) were highest in study participants that experienced positive impact of the pandemic on their lifestyle (family relations, financial security, diet, sleep habits, health status) or no impact associated with the pandemic (alcohol consumption, social interactions, physical activity). There were similar shares of flourishing individuals both with and without pre-existing chronic conditions. Individuals with pre-existing mental disorder and those that were exposed to violence during the pandemic were flourishing in substantially lower shares relative to individuals without those exposures.

**Table 3:** Prevalence and results of regression analysis for flourishing mental health.

		**Unadjusted OR**		**Adjusted model 1**		**Adjusted model 2**	
	**%**	**OR (95% CI)**	***p***	**OR (95% CI)**	***p***	**OR (95% CI)**	***p***
**Sex**							
Male	40.5	1.19 (1.04–1.36)	0.014	1.17 (0.99–1.37)	0.060	1.00 (0.84–1.20)	0.959
Female	36.6	REF		REF		REF	
**Age (years)**							
18–24	30.3	REF		REF		REF	
25–34	32.1	1.09 (0.80–1.47)	0.588	0.62 (0.39–0.99)	0.047	0.50 (0.30–0.84)	0.008
35–44	41.8	1.64 (1.23–2.19)	0.001	0.87 (0.53–1.41)	0.565	0.71 (0.41–1.22)	0.211
45–54	36.5	1.33 (0.99–1.78)	0.061	0.67 (0.41–1.10)	0.114	0.53 (0.30–0.92)	0.024
55–64	43.3	1.74 (1.30–2.33)	<0.001	0.69 (0.41–1.16)	0.164	0.58 (0.32–1.04)	0.066
65–74	42.5	1.64 (1.20–2.25)	0.002	0.49 (0.26–0.92)	0.026	0.40 (0.20–0.81)	0.011
75 and more	40.6	1.53 (1.10–2.14)	0.013	0.47 (0.24–0.93)	0.03	0.41 (0.19–0.86)	0.019
**Education status**							
Primary school or lower	34.3	REF		REF		REF	
Secondary school	39.4	1.27 (1.05–1.53)	0.016	1.43 (1.13–1.82)	0.003	1.49 (1.14–1.95)	0.003
College or higher	39.8	1.29 (1.05–1.60)	0.018	1.49 (1.14–1.95)	0.004	1.47 (1.09–1.98)	0.012
**Employment status**							
Employed, self–employed	38.1	REF		REF		REF	
Student	28.5	0.65 (0.50–0.85)	0.002	0.76 (0.47–1.22)	0.250	0.77 (0.46–1.31)	0.338
Retired	44.6	1.27 (1.08–1.49)	0.003	1.68 (1.18–2.40)	0.004	1.82 (1.23–2.69)	0.003
Unemployed	29.8	0.70 (0.51–0.94)	0.018	0.86 (0.61–1.22)	0.398	1.02 (0.70–1.50)	0.908
**Marital status**							
Married	44.3	REF		REF		REF	
Civil partner	39.0	0.80 (0.67–0.95)	0.010	0.83 (0.67–1.01)	0.069	0.93 (0.74–1.16)	0.514
Single	27.1	0.47 (0.39–0.57)	<0.001	0.48 (0.37–0.63)	<0.001	0.48 (0.36–0.64)	<0.001
Widowed	36.2	0.68 (0.50–0.92)	0.011	0.79 (0.53–1.19)	0.258	0.86 (0.55–1.35)	0.511
Divorced	32.5	0.60 (0.40–0.91)	0.017	0.58 (0.36–0.93)	0.025	0.64 (0.38–1.07)	0.087
**Alcohol consumption**							
Decreased	36.0	0.88 (0.74–1.06)	0.173			0.84 (0.54–1.30)	0.430
Unchanged	39.0	REF				REF	
Increased	30.7	0.69 (0.48–0.99)	0.043			0.77 (0.48–1.24)	0.287
**COVID−19 infection**							
Yes	39.1	REF				REF	
No	38.1	0.96 (0.79–1.16)	0.653			1.02 (0.81–1.29)	0.833
**Family relations**							
Worsened	17.7	0.32 (0.25–0.41)	<0.001			0.61 (0.45–0.83)	0.001
Unchanged	40.2	REF				REF	
Improved	48.7	1.41 (1.19–1.67)	<0.001			1.35 (1.08–1.68)	0.007
**Financial security**							
Worsened	31.0	0.64 (0.55–0.75)	<0.001			1.15 (0.93–1.41)	0.192
Unchanged	43.2	REF				REF	
Improved	48.8	1.36 (1.07–1.73)	0.013			1.47 (1.07–2.01)	0.016
**Social interactions**							
Worsened	34.1	0.51 (0.43–0.60)	<0.001			0.66 (0.54–0.82)	<0.001
Unchanged	50.4	REF				REF	
Improved	48.9	0.95 (0.66–1.35)	0.763			0.89 (0.56–1.40)	0.605
**Physical activity**							
Worsened	29.4	0.49 (0.42–0.57)	<0.001			0.78 (0.63–0.95)	0.016
Unchanged	46.0	REF				REF	
Improved	44.9	0.96 (0.78–1.17)	0.676			0.78 (0.58–1.03)	0.083
**Diet**							
Worsened	20.3	0.36 (0.29–0.44)	<0.001			0.61 (0.46–0.82)	0.001
Unchanged	41.7	REF				REF	
Improved	45.6	1.17 (0.97–1.41)	0.101			1.06 (0.80–1.41)	0.698
**Sleep**							
Worsened	21.5	0.35 (0.29–0.41)	<0.001			0.66 (0.52–0.84)	0.001
Unchanged	44.2	REF				REF	
Improved	48.7	1.20 (0.97–1.49)	0.093			1.12 (0.83–1.51)	0.441
**Health status**							
Worsened	23.4	0.42 (0.35–0.51)	<0.001			0.88 (0.68–1.14)	0.327
Unchanged	42.0	REF				REF	
Improved	49.6	1.37 (0.94–2.00)	0.099			1.26 (0.77–2.07)	0.360
**Pre–existing chronic conditions**							
Yes	38.2	REF				REF	
No	39.2	1.04 (0.90–1.21)	0.574			0.99 (0.81–1.21)	0.901
**Pre–existing mental disorders**							
Yes	18.2	REF				REF	
No	39.8	2.99 (2.06–4.35)	<0.001			1.89 (1.19–3.00)	0.007
**Exposure to violence**							
Yes	22.3	REF				REF	
No	40.4	2.38 (1.84–3.08)	<0.001			1.42 (1.03–1.95)	0.033
**Resilience**	/	1.42 (1.35–1.48)	<0.001			1.34 (1.26–1.41)	<0.001
**Literacy; finding, understanding and evaluating information**	/	1.36 (1.28–1.46)	<0.001			1.11 (1.01–1.22)	0.023
**Literacy; understanding and following recommendations**	/	1.29 (1.21–1.37)	<0.001			1.18 (1.08–1.30.)	<0.001

#### Socio-demographic factors

In the adjusted model, having secondary education or higher was associated with higher odds of having flourishing mental health compared to primary education or lower. Being retired showed increased odds of flourishing mental health compared to being employed or self-employed. Being single was significantly associated with decreased odds of flourishing mental health relative to being married.

#### Health and health behavior related factors

Having pre-existent chronic conditions (diagnosed before the pandemic) was not significantly associated with odds of flourishing mental health. However, not having a pre-existent mental disorder showed increased odds of flourishing mental health. Resilience and health literacy measures were significantly associated with flourishing mental health.

Those who assessed that the pandemic worsened their physical activity, diet and sleep habits showed decreased odds of flourishing mental health in comparison to those whose habits were unaffected by the pandemic.

Worsened family relations and social interactions showed decreased odds of flourishing mental health. Not being exposed to violence and improved financial security were associated with increased odds of flourishing mental health.

### Languishing mental health during COVID-19

Shares of individuals with languishing mental health (Table 4) were highest in study participants that experienced negative impact of the pandemic on their lifestyle (that held true for all included factors). There were no significant differences in shares of individuals with languishing mental health in relation to pre-existing chronic conditions or COVID-19 infection status. However, there was a substantial difference in languishing mental health between people that were diagnosed with a mental disorder before the pandemic (22.4 %) in comparison to individuals free of mental disorder diagnosis (7.2 %).

**Table 4:** Prevalence and results of regression analysis for languishing mental health.

		**Unadjusted OR**		**Adjusted model 1**		**Adjusted model 2**	
	**%**	**OR (95% CI)**	***p***	**OR (95% CI)**	***p***	**OR (95% CI)**	***p***
**Sex**							
Male	6.6	REF		REF		REF	
Female	9.4	1.47 (1.15–1.89)	0.002	1.47 (1.1–1.97)	0.010	1.14 (0.82–1.58)	0.443
**Age (years)**							
18–24	14.9	REF		REF		REF	
25–34	12.9	0.84 (0.56–1.26)	0.404	1.56 (0.84–2.91)	0.163	2.00 (0.99–4.04)	0.054
35–44	6.9	0.43 (0.28–0.66)	<0.001	0.88 (0.43–1.79)	0.720	1.27 (0.57–2.86)	0.559
45–54	5.3	0.32 (0.20–0.52)	<0.001	0.82 (0.39–1.71)	0.592	1.10 (0.48–2.53)	0.817
55–64	5.2	0.31 (0.19–0.50)	<0.001	0.98 (0.44–2.17)	0.956	1.22 (0.49–3.01)	0.668
65–74	5.8	0.35 (0.21–0.59)	<0.001	1.23 (0.4–3.75)	0.721	1.73 (0.50–5.99)	0.390
75 and more	8.7	0.55 (0.33–0.91)	0.020	2.03 (0.64–6.43)	0.227	2.57 (0.72–9.18)	0.146
**Education status**							
Primary school or lower	9.7	REF		REF		REF	
Secondary school	7.8	0.79 (0.57–1.08)	0.136	1.01 (0.68–1.51)	0.961	1.14 (0.73–1.78)	0.555
College or higher	7.2	0.72 (0.50–1.04)	0.083	0.87 (0.54–1.39)	0.554	1.11 (0.66–1.86)	0.699
**Employment status**							
Employed, self–employed	6.3	REF		REF		REF	
Student	15.6	2.77 (1.92–4.00)	<0.001	1.64 (0.88–3.05)	0.118	2.28 (1.14–4.55)	0.02
Retired	6.1	0.97 (0.70–1.34)	0.842	0.71 (0.32–1.58)	0.400	0.65 (0.27–1.52)	0.317
Unemployed	18.8	3.48 (2.37–5.10)	<0.001	2.60 (1.67–4.05)	<0.001	2.55 (1.52–4.28)	<0.001
**Marital status**							
Married	4.6	REF		REF		REF	
Civil partner	7.6	1.75 (1.23–2.47)	0.002	1.56 (1.01–2.42)	0.044	1.30 (0.81–2.08)	0.277
Single	14.9	3.69 (2.69–5.06)	<0.001	3.10 (2–4.82)	<0.001	2.77 (1.73–4.43)	<0.001
Widowed	10.2	2.43 (1.47–4.01)	0.001	1.77 (0.85–3.7)	0.13	1.53 (0.71–3.29)	0.278
Divorced	11.5	2.73 (1.45–5.14)	0.002	3.03 (1.49–6.16)	0.002	2.60 (1.19–5.67)	0.017
**Alcohol consumption**							
Decreased	6.7	0.83 (0.59–1.17)	0.287			0.62 (0.40–0.96)	0.032
Unchanged	7.9	REF				REF	
Increased	16.7	2.34 (1.49–3.68)	<0.001			1.13 (0.63–2.00)	0.687
**COVID−19 infection**							
Yes	7.4	1.14 (0.81–1.61)	0.450			1.02 (0.67–1.54)	0.941
No	8.4	REF				REF	
**Family relations**							
Worsened	17.9	2.97 (2.25–3.91)	<0.001			1.57 (1.06–2.31)	0.023
Unchanged	6.9	REF				REF	
Improved	4.4	0.63 (0.43–0.93)	0.021			0.91 (0.57–1.44)	0.673
**Financial security**							
Worsened	12.6	2.38 (1.84–3.08)	<0.001			1.10 (0.78–1.56)	0.593
Unchanged	5.7	REF				REF	
Improved	5.7	1.01 (0.60–1.70)	0.955			1.21 (0.65–2.28)	0.547
**Social interactions**							
Worsened	8.9	1.48 (1.07–2.03)	0.016			0.76 (0.51–1.15)	0.200
Unchanged	6.2	REF				REF	
Improved	4.3	0.64 (0.27–1.55)	0.325			0.24 (0.07–0.85)	0.027
**Physical activity**							
Worsened	12.0	2.45 (1.85–3.24)	<0.001			1.14 (0.77–1.69)	0.509
Unchanged	5.3	REF				REF	
Improved	4.2	0.79 (0.49–1.28)	0.341			0.67 (0.36–1.26)	0.214
**Diet**							
Worsened	19.0	3.75 (2.87–4.90)	<0.001			1.87 (1.27–2.74)	0.002
Unchanged	5.9	REF				REF	
Improved	5.2	0.86 (0.57–1.30)	0.477			0.72 (0.40–1.31)	0.286
**Sleep**							
Worsened	16.1	3.77 (2.90–4.92)	<0.001			1.40 (0.97–2.02)	0.075
Unchanged	4.8	REF				REF	
Improved	5.8	1.23 (0.77–1.95)	0.384			1.20 (0.64–2.22)	0.570
**Health status**							
Worsened	16.5	3.09 (2.39–4.00)	<0.001			1.50 (1.06–2.14)	0.024
Unchanged	6.0	REF				REF	
Improved	5.3	0.85 (0.36–1.99)	0.700			0.76 (0.21–2.70)	0.668
**Pre–existing chronic conditions**							
Yes	8.1	1.01 (0.78–1.32)	0.918			1.01 (0.70–1.46)	0.961
No	7.9	REF				REF	
**Pre–existing mental disorders**							
Yes	22.4	3.70 (2.57–5.34)	<0.001			1.60 (0.98–2.62)	0.062
No	7.2	REF				REF	
**Exposure to violence**							
Yes	17.6	2.90 (2.14–3.93)	<0.001			1.33 (0.88–2.01)	0.172
No	6.8	REF				REF	
**Resilience**	/	0.64 (0.59–0.69)	<0.001			0.72 (0.65–0.80)	<0.001
**Literacy; finding, understanding and evaluating information**	/	0.70 (0.64–0.77)	<0.001			0.78 (0.68–0.90)	8E−04
**Literacy; understanding and following recommendations**	/	0.79 (0.72–0.86)	<0.001			1.05 (0.91–1.21)	0.511

#### Socio-demographic factors

Students and unemployed showed increased odds of languishing mental health in comparison to employed and self-employed. Those who were single or divorced had higher odds of languishing mental health relative to married individuals.

#### Health and health behavior related factors

Neither pre-existing chronic conditions nor mental disorders showed significant association with odds of languishing mental health in the adjusted model. Those who experienced worsening of their health status during the pandemic had increased odds of languishing mental health compared to individuals without changes in health status. Negative impact of the pandemic on the diet was also associated with increased odds of languishing health relative to no impact on the diet. Decreased alcohol consumption, resilience and literacy, defined as a capability to find, understand and evaluate information related to COVID-19, were inversely related to odds of languishing mental health.

Worsened family relations were associated with increased odds of languishing mental health in comparison to unchanged family relations. Those who improved their social interactions during COVID-19 pandemic showed decreased odds of languishing mental health in comparison to unchanged social interactions.

## Discussion

### Positive mental health before and during COVID-19 pandemic

Findings from two cross-sectional, nationally representative surveys from before and during the pandemic showed substantial decrease in the share of the population in flourishing mental health and an increase in the share of the population in languishing mental health.

The distribution of PMH presented in this article is comparable to other estimates obtained during the pandemic. Gloster et al. included nearly 10,000 participants from 78 countries, including Slovenia, in their research (6). They found 10.1% experience languishing and 39,9% flourishing mental health, while we estimated 8.0% (7.1–8.9%) and 38.6% (37.0–40.2%) experience languishing and flourishing mental health, respectively. Cross-cultural comparisons on the MHC-SF results show substantial variation in prevalence rates in different high-income countries. Pre-pandemic estimates on the prevalence of languishing mental health ranged from 0.9 to 3.9%, and from 38.6 to 82.8% for flourishing mental health (15). Direct comparison of PMH prevalence rates, obtained by a single cross-sectional study, is therefore not optimal. Repeated cross-sectional or longitudinal studies would offer a better insight into PMH distribution and changes within the country. Such knowledge would help to contextualize estimates and make international comparisons more feasible.

Few studies compared PMH before and during the pandemic on a general population samples (12, 16, 17). Majority of studies during the pandemic show initial deterioration of PMH (18). Direct comparison with results presented in this article is not possible because of different instruments used to measure PMH. Some studies, limited to specific population groups, such as social workers in McFadden et al. or older adults in Hansen et al. found mental well-being increased or remained stable relative to before pandemic (19, 20). The maintained or improved level of PMH is attributed to population-specific factors that are not pervasive in the general population, and to the phase of the pandemic during which research took place, highlighting the accumulation and intensification of negative psychosocial experiences that results in deterioration of PMH in later stages of the pandemic (18, 19). In contrast, first months after the crisis event, first wave of the pandemic for example, are described as heroic and honeymoon phases of emotional response (21). Although there are notable differences in the dynamics of the COVID-19 pandemic and other one-time crisis events such as earthquakes or floods, we might attribute stable or even improved mental well-being to the specific emotional response in the 1st months of the pandemic when majority of the published research took place.

### COVID-19 pandemic and its impact on flourishing mental health

Findings from our study are in large part confirmatory to other research on the impact of COVID-19 pandemic on mental health. The odds of flourishing mental health follow the socio-economic gradient, with higher odds of flourishing mental health among more educated and among employed relative to unemployed or those still in education. This was an expected result, as a number of studies have reached the similar conclusion both before and during the pandemic (6, 9, 22, 23). From changes in health-related behavior that COVID-19 has caused, only improved family relations and financial security were significantly associated with increased odds of flourishing mental health, adding to the importance of family resilience during times when in-person social interactions are limited to a single household (24). We have collected data at a peak of the second COVID-19 wave in Slovenia, during which we experienced highest case and death rates. During this time, various uncertainties peaked and those who managed to improve their financial outlooks fared better in many aspects of PMH. Concurrently strict measures to limit the spread of the virus were imposed, restricting majority of social interactions bar those within family, in selected workplaces and over the internet. This might also explain the increased odds of flourishing mental health we identified when family relations were improved compared to unchanged.

No other improvement in factors affected by the pandemic was significantly associated with increased odds of flourishing mental health. Prevalence of flourishing mental health was similar in individuals who experienced no change or improvement in majority of factors liable to COVID-19 pandemic. Since we did not control for pre-pandemic levels of those factors, a ceiling effect might occur when one's health-related and social factors were already at optimal level. This might be the cause for the absence of significant differences in flourishing mental health between participants who reported no change and those who reported improvement in specific factors. Therefore, flourishing mental health was better predicted by sociodemographic variables, freedom from violence and absence of mental disorder. Self-reporting unchanged status might also signify a sense of normality that contributes to PMH during tumultuous times of the pandemic. Gloster et al. for example found that people who left their house more often during the pandemic experienced more positive affect, possibly due to more variation in everyday life and consequently greater sense of normality (6).

Both resilience and health literacy related to COVID-19 pandemic information and recommendations also showed significantly increased odds of flourishing mental health. Substantial association of resilience and flourishing mental health was established also by Kavčič et al. (7) who highlight the importance of subjective perception of one's health in comparison to objective health indicators such as presence of a chronic health condition. Indeed, our results lead us to the same conclusion, as neither pre-existing chronic condition neither COVID-19 infection showed significant association with the odds of flourishing mental health. However, this also might be due to selection bias since those that suffered from more severe forms of COVID-19 might not be able or willing to participate in our research.

### COVID-19 pandemic and its impact on languishing mental health

Contrary to other research on poor PMH, we did not find significant associations between education and languishing mental health during the pandemic (4, 23). However, being a student or unemployed relative to employer or self-employed and being single or divorced relative to married showed increased odds of languishing mental health. These factors are related to financial insecurity, social isolation and other social determinants of mental health, for most of which we did not establish a baseline in our study (25). Hence, low levels of financial security, social interactions or physical activity, that are associated with low PMH and did not change during the pandemic, might have been unnoticed in our analysis.

We measured changes perceived by study participants in a number of social determinants, but interestingly, we only found increased odds of languishing mental health with worsened health status, family relations and diet – all compared to unchanged by the pandemic. Food insecurity has been linked with poor mental health in recent research and our results corroborate the importance of this often overlooked aspect in mental health research (26, 27). An alternative interpretation of the association of worsened diet and languishing mental health is related to emotional eating triggered by the pandemic events (28). Perhaps even more surprising was absence of statistically significant association of exposure to violence or pre-existing mental disorders on languishing mental health. Both factors were significantly associated with poor PMH in the univariate analysis but have lost the level of statistical significance in the full regression model. This indicates the experience of languishing mental health is better predicted by reported changes in health-related measures or unmeasured factors associated with employment and marital status.

### Implications for the public health practice

Mental health is one of the most exposed topics when discussing the impacts of COVID-19 pandemic. Undoubtedly, the pandemic has had and will continue to have an important impact on the state of mental health of the population, further influencing not only mental health issues but other health related behaviors as well. However, the discourse is primarily disorder oriented, with symptoms of depression, anxiety disorders, self-harm, and stress disorders (29). With our research we add to the understudied area of the impact of the pandemic on PMH. We show important changes in the levels of PMH on a nationally representative sample from Slovenia. Studies have shown PMH is related to the occurrence of chronic disorders, mental health conditions included, and is therefore an important factor contributing to the burden of disease associated with the pandemic (30). Public health efforts need to accommodate both prevention of mental health conditions and mental health promotion. Both approaches need to be considered to address complete public mental health needs. Even though there is plenty of evidence on effective mental health interventions, we fail to implement them on a sufficient scale to impact population mental health (31). Drawing from our study, special attention needs to be placed on social aspects of well-being. Significant associations of family relations and social interactions with PMH highlight the need to address and further investigate the impact of the pandemic on these areas. Food insecurity and mental health is another area of significance, especially in the future when prices are expected to rise even more due to war in Ukraine (32). Areas where public health interventions could achieve multiplicative effects are resilience and health literacy. We found significant associations for higher odds of flourishing mental health and lower odds of languishing mental health for both concepts in our study, while other studies show improved health outcomes in a number of other areas (33, 34).

### Limitations

There are a number of limitations to the study that need to be considered. First is the cross-sectional nature of the research, due to which causal relations cannot be interpreted from the data. We have included a series of questions to quantify the impact of the pandemic, however we did not include retrospective questions to establish a baseline for each of the areas we assessed the impact on. The samples in both surveys used in the research are general household samples and do not include people with significant disabilities. Therefore, the most vulnerable population that was also heavily impacted by the pandemic is not represented in our data.

## Data availability statement

The data analyzed in this study is subject to the following licenses/restrictions: Datasets were compiled with research conducted by the National Institute of Public Health. Datasets are not available publicly. They can be acquired by registered researchers and research institutions. Requests to access these datasets should be directed to statisticna.pisarna@nijz.si.

## Author contributions

MV, PM, SR, and HJ contributed to conception and design of the study and wrote sections of the manuscript. PM organized the database. PM and MV performed the statistical analysis. MV wrote the first draft of the manuscript. All authors contributed to manuscript revision, read, and approved the submitted version.

## Funding

The National Institute of Public Health of Slovenia has funded open access (APC) for this publication.

## Conflict of interest

The authors declare that the research was conducted in the absence of any commercial or financial relationships that could be construed as a potential conflict of interest.

## Publisher's note

All claims expressed in this article are solely those of the authors and do not necessarily represent those of their affiliated organizations, or those of the publisher, the editors and the reviewers. Any product that may be evaluated in this article, or claim that may be made by its manufacturer, is not guaranteed or endorsed by the publisher.
