# Supplementary material for: Positive mental health in Slovenia before and during the COVID-19 pandemic
Source: Front Public Health. 2022 Oct 14;10:963545. doi: 10.3389/fpubh.2022.963545 (PMC9614317; doi:10.3389/fpubh.2022.963545)
Supplement: Supplementary file 1 [file Table_1.DOCX]

Supplementary Material

# Appendix 1: Sociodemographic characteristics of study participants

Table 1: Table on socio-demographic characteristics of EHIS and SI-PANDA samples

|  | **EHIS 2019** | | | **PANDA 2021** | | |
| --- | --- | --- | --- | --- | --- | --- |
|  | Unweighted | | Weighted | Unweighted | | Weighted |
|  | N | % | % | N | % | % |
| **Sex** | | | | | | |
| male | 4059 | 44,9 | 49,4 | 1617 | 46,7 | 51,0 |
| female | 4988 | 55,1 | 50,6 | 1845 | 53,3 | 49,0 |
| **Age** |  |  |  |  |  |  |
| 18 - 24 | 785 | 8,7 | 8,4 | 245 | 7,1 | 8,7 |
| 25 - 34 | 1089 | 12,0 | 15,1 | 399 | 11,6 | 15,7 |
| 35 - 44 | 1545 | 17,1 | 18,6 | 520 | 14,9 | 19,2 |
| 45 - 54 | 1614 | 17,8 | 17,7 | 570 | 16,5 | 18,2 |
| 55 - 64 | 1679 | 18,5 | 17,4 | 748 | 21,6 | 17,3 |
| 65 - 74 | 1344 | 14,9 | 12,9 | 592 | 17,1 | 12,0 |
| 75 and more | 991 | 11,0 | 9,9 | 388 | 11,2 | 8,9 |
| **Marital status** | | | | | | |
| married | 4672 | 51,9 | 50,2 | 1732 | 50,3 | 45,3 |
| civil partner | 1253 | 13,9 | 15,0 | 764 | 22,0 | 24,7 |
| single | 1755 | 19,5 | 21,0 | 573 | 16,7 | 20,7 |
| widowed | 812 | 9,0 | 8,5 | 256 | 7,3 | 6,1 |
| divorced | 516 | 5,7 | 5,3 | 126 | 3,7 | 3,2 |
| **Education status** | | | | | | |
| primary education or lower | 1264 | 14,0 | 19,5 | 419 | 12,1 | 17,8 |
| secondary education | 4963 | 54,8 | 54,7 | 1742 | 50,3 | 55,0 |
| college or higher | 2820 | 31,2 | 25,8 | 1301 | 37,6 | 27,2 |
| **Employment status** | | | | | | |
| employed, self-employed | 4609 | 51,2 | 53,8 | 1763 | 51,0 | 56,7 |
| student | 576 | 6,4 | 6,1 | 239 | 6,9 | 8,3 |
| retired | 3045 | 33,8 | 30,4 | 1201 | 34,6 | 26,2 |
| unemployed | 507 | 5,6 | 6,4 | 178 | 5,2 | 6,4 |
| other | 265 | 3,0 | 3,3 | 77 | 2,3 | 2,4 |

# Appendix 2: Results of univariate and multinomial regression analysis for flourishing and languishing mental health

Table 1: Results of regression analysis for flourishing mental health.

| **Flourishing mental health** | | | | | |
| --- | --- | --- | --- | --- | --- |
|  |  | **Unadjusted model** | | **Adjusted model** | |
|  | **%** | OR (95% CI) | *p* | OR (95% CI) | *p* |
| **Sex** | | | | | |
| male | 40,5 | 1,19 (1,04–1,36) | 0,014 | 1,00 (0,84-1,20) | 0,959 |
| female | 36,6 | REF |  | REF |  |
| **Age** | | | | | |
| 18 - 24 | 30,3 | REF |  | REF |  |
| 25 - 34 | 32,1 | 1,09 (0,80–1,47) | 0,588 | 0,50 (0,30-0,84) | 0,008 |
| 35 - 44 | 41,8 | 1,64 (1,23–2,19) | 0,001 | 0,71 (0,41-1,22) | 0,211 |
| 45 - 54 | 36,5 | 1,33 (0,99–1,78) | 0,061 | 0,53 (0,30-0,92) | 0,024 |
| 55 - 64 | 43,3 | 1,74 (1,30–2,33) | <0,001 | 0,58 (0,32-1,04) | 0,066 |
| 65 - 74 | 42,5 | 1,64 (1,20–2,25) | 0,002 | 0,40 (0,20-0,81) | 0,011 |
| 75 and more | 40,6 | 1,53 (1,10–2,14) | 0,013 | 0,41 (0,19-0,86) | 0,019 |
| **Education status** | | | | | |
| primary school or lower | 34,3 | REF |  | REF |  |
| secondary school | 39,4 | 1,27 (1,05–1,53) | 0,016 | 1,49 (1,14–1,95) | 0,003 |
| college or higher | 39,8 | 1,29 (1,05–1,60) | 0,018 | 1,47 (1,09–1,98) | 0,012 |
| **Employment status** | | | | | |
| employed, self-employed | 38,1 | REF |  | REF |  |
| student | 28,5 | 0,65 (0,50–0,85) | 0,002 | 0,77 (0,46–1,31) | 0,338 |
| retired | 44,6 | 1,27 (1,08–1,49) | 0,003 | 1,82 (1,23–2,69) | 0,003 |
| unemployed | 29,8 | 0,70 (0,51–0,94) | 0,018 | 1,02 (0,70–1,50) | 0,908 |
| **Marital status** | | | | | |
| married | 44,3 | REF |  | REF |  |
| civil partner | 39,0 | 0,80 (0,67–0,95) | 0,010 | 0,93 (0,74–1,16) | 0,514 |
| single | 27,1 | 0,47 (0,39–0,57) | <0,001 | 0,48 (0,36–0,64) | <0,001 |
| widowed | 36,2 | 0,68 (0,50–0,92) | 0,011 | 0,86 (0,55–1,35) | 0,511 |
| divorced | 32,5 | 0,60 (0,40–0,91) | 0,017 | 0,64 (0,38–1,07) | 0,087 |
| **Alcohol consumption** | | | | | |
| Decreased | 36,0 | 0,88 (0,74–1,06) | 0,173 | 0,84 (0,54–1,30) | 0,43 |
| Unchanged | 39,0 | REF |  | REF |  |
| Increased | 30,7 | 0,69 (0,48–0,99) | 0,043 | 0,77 (0,48–1,24) | 0,287 |
| **COVID-19 infection** | | | | | |
| Yes | 39,1 | REF |  | REF |  |
| No | 38,1 | 0,96 (0,79–1,16) | 0,653 | 1,02 (0,81–1,29) | 0,833 |
| **Family relations** | | | | | |
| Worsened | 17,7 | 0,32 (0,25–0,41) | <0,001 | 0,61 (0,45–0,83) | 0,001 |
| Unchanged | 40,2 | REF |  | REF |  |
| Improved | 48,7 | 1,41 (1,19–1,67) | <0,001 | 1,35 (1,08–1,68) | 0,007 |
| **Financial security** | | | | | |
| Worsened | 31,0 | 0,64 (0,55–0,75) | <0,001 | 1,15 (0,93–1,41) | 0,192 |
| Unchanged | 43,2 | REF |  | REF |  |
| Improved | 48,8 | 1,36 (1,07–1,73) | 0,013 | 1,47 (1,07–2,01) | 0,016 |
| **Social interactions** | | | | | |
| Worsened | 34,1 | 0,51 (0,43–0,60) | <0,001 | 0,66 (0,54–0,82) | <0,001 |
| Unchanged | 50,4 | REF |  | REF |  |
| Improved | 48,9 | 0,95 (0,66–1,35) | 0,763 | 0,89 (0,56–1,40) | 0,605 |
| **Physical activity** | | | | | |
| Worsened | 29,4 | 0,49 (0,42–0,57) | <0,001 | 0,78 (0,63–0,95) | 0,016 |
| Unchanged | 46,0 | REF |  | REF |  |
| Improved | 44,9 | 0,96 (0,78–1,17) | 0,676 | 0,78 (0,58–1,03) | 0,083 |
| **Diet** | | | | | |
| Worsened | 20,3 | 0,36 (0,29–0,44) | <0,001 | 0,61 (0,46–0,82) | 0,001 |
| Unchanged | 41,7 | REF |  | REF |  |
| Improved | 45,6 | 1,17 (0,97–1,41) | 0,101 | 1,06 (0,80–1,41) | 0,698 |
| **Sleep** | | | | | |
| Worsened | 21,5 | 0,35 (0,29–0,41) | <0,001 | 0,66 (0,52–0,84) | 0,001 |
| Unchanged | 44,2 | REF |  | REF |  |
| Improved | 48,7 | 1,20 (0,97–1,49) | 0,093 | 1,12 (0,83–1,51) | 0,441 |
| **Health status** | | | | | |
| Worsened | 23,4 | 0,42 (0,35–0,51) | <0,001 | 0,88 (0,68–1,14) | 0,327 |
| Unchanged | 42,0 | REF |  | REF |  |
| Improved | 49,6 | 1,37 (0,94–2,00) | 0,099 | 1,26 (0,77–2,07) | 0,36 |
| **Pre-existing chronic conditions** | | | | | |
| Yes | 38,2 | REF |  | REF |  |
| No | 39,2 | 1,04 (0,90–1,21) | 0,574 | 0,99 (0,81–1,21) | 0,901 |
| **Pre-existing mental disorders** | | | | | |
| Yes | 18,2 | REF |  | REF |  |
| No | 39,8 | 2,99 (2,06–4,35) | <0,001 | 1,89 (1,19–3,00) | 0,007 |
| **Exposure to violence** | | | | | |
| Yes | 22,3 | REF |  | REF |  |
| No | 40,4 | 2,38 (1,84–3,08) | <0,001 | 1,42 (1,03–1,95) | 0,033 |
| **Resilience** | / | 1,42 (1,35–1,48) | <0,001 | 1,34 (1,26–1,41) | <0,001 |
| **Literacy; finding, understanding and evaluating information** | / | 1,36 (1,28–1,46) | <0,001 | 1,11 (1,01–1,22) | 0,023 |
| **Literacy; understanding and following recommendations** | / | 1,29 (1,21–1,37) | <0,001 | 1,18 (1,08–1,30) | <0,001 |

Table 2: Multinomial regression models for flourishing mental health.

| **Multinomial regression models** | | | | | |
| --- | --- | --- | --- | --- | --- |
|  |  | **Model 1** | | **Model 3** | |
|  | **%** | OR (95% CI) | *p* | OR (95% CI) | *p* |
| **Sex** | | | | | |
| male | 40,5 | 1,17 (0,99–1,37) | 0,060 | 1,00 (0,84-1,20) | 0,959 |
| female | 36,6 | REF |  | REF |  |
| **Age** | | | | | |
| 18 - 24 | 30,3 | REF |  | REF |  |
| 25 - 34 | 32,1 | 0,62 (0,39–0,99) | 0,047 | 0,50 (0,30-0,84) | 0,008 |
| 35 - 44 | 41,8 | 0,87 (0,53–1,41) | 0,565 | 0,71 (0,41-1,22) | 0,211 |
| 45 - 54 | 36,5 | 0,67 (0,41–1,10) | 0,114 | 0,53 (0,30-0,92) | 0,024 |
| 55 - 64 | 43,3 | 0,69 (0,41–1,16) | 0,164 | 0,58 (0,32-1,04) | 0,066 |
| 65 - 74 | 42,5 | 0,49 (0,26–0,92) | 0,026 | 0,40 (0,20-0,81) | 0,011 |
| 75 and more | 40,6 | 0,47 (0,24–0,93) | 0,03 | 0,41 (0,19-0,86) | 0,019 |
| **Education status** | | | | | |
| primary school or lower | 34,3 | REF |  | REF |  |
| secondary school | 39,4 | 1,43 (1,13–1,82) | 0,003 | 1,49 (1,14–1,95) | 0,003 |
| college or higher | 39,8 | 1,49 (1,14–1,95) | 0,004 | 1,47 (1,09–1,98) | 0,012 |
| **Employment status** | | | | | |
| employed, self-employed | 38,1 | REF |  | REF |  |
| student | 28,5 | 0,76 (0,47–1,22) | 0,250 | 0,77 (0,46–1,31) | 0,338 |
| retired | 44,6 | 1,68 (1,18–2,40) | 0,004 | 1,82 (1,23–2,69) | 0,003 |
| unemployed | 29,8 | 0,86 (0,61–1,22) | 0,398 | 1,02 (0,70–1,50) | 0,908 |
| **Marital status** | | | | | |
| married | 44,3 | REF |  | REF |  |
| civil partner | 39,0 | 0,83 (0,67–1,01) | 0,069 | 0,93 (0,74–1,16) | 0,514 |
| single | 27,1 | 0,48 (0,37–0,63) | <0,001 | 0,48 (0,36–0,64) | <0,001 |
| widowed | 36,2 | 0,79 (0,53–1,19) | 0,258 | 0,86 (0,55–1,35) | 0,511 |
| divorced | 32,5 | 0,58 (0,36–0,93) | 0,025 | 0,64 (0,38–1,07) | 0,087 |
| **Alcohol consumption** | | | | | |
| Decreased | 36,0 |  |  | 0,84 (0,54–1,30) | 0,43 |
| Unchanged | 39,0 |  |  | REF |  |
| Increased | 30,7 |  |  | 0,77 (0,48–1,24) | 0,287 |
| **COVID-19 infection** | | | | | |
| Yes | 39,1 |  |  | REF |  |
| No | 38,1 |  |  | 1,02 (0,81–1,29) | 0,833 |
| **Family relations** | | | | | |
| Worsened | 17,7 |  |  | 0,61 (0,45–0,83) | 0,001 |
| Unchanged | 40,2 |  |  | REF |  |
| Improved | 48,7 |  |  | 1,35 (1,08–1,68) | 0,007 |
| **Financial security** | | | | | |
| Worsened | 31,0 |  |  | 1,15 (0,93–1,41) | 0,192 |
| Unchanged | 43,2 |  |  | REF |  |
| Improved | 48,8 |  |  | 1,47 (1,07–2,01) | 0,016 |
| **Social interactions** | | | | | |
| Worsened | 34,1 |  |  | 0,66 (0,54–0,82) | <0,001 |
| Unchanged | 50,4 |  |  | REF |  |
| Improved | 48,9 |  |  | 0,89 (0,56–1,40) | 0,605 |
| **Physical activity** | | | | | |
| Worsened | 29,4 |  |  | 0,78 (0,63–0,95) | 0,016 |
| Unchanged | 46,0 |  |  | REF |  |
| Improved | 44,9 |  |  | 0,78 (0,58–1,03) | 0,083 |
| **Diet** | | | | | |
| Worsened | 20,3 |  |  | 0,61 (0,46–0,82) | 0,001 |
| Unchanged | 41,7 |  |  | REF |  |
| Improved | 45,6 |  |  | 1,06 (0,80–1,41) | 0,698 |
| **Sleep** | | | | | |
| Worsened | 21,5 |  |  | 0,66 (0,52–0,84) | 0,001 |
| Unchanged | 44,2 |  |  | REF |  |
| Improved | 48,7 |  |  | 1,12 (0,83–1,51) | 0,441 |
| **Health status** | | | | | |
| Worsened | 23,4 |  |  | 0,88 (0,68–1,14) | 0,327 |
| Unchanged | 42,0 |  |  | REF |  |
| Improved | 49,6 |  |  | 1,26 (0,77–2,07) | 0,36 |
| **Pre-existing chronic conditions** | | | | | |
| Yes | 38,2 |  |  | REF |  |
| No | 39,2 |  |  | 0,99 (0,81–1,21) | 0,901 |
| **Pre-existing mental disorders** | | | | | |
| Yes | 18,2 |  |  | REF |  |
| No | 39,8 |  |  | 1,89 (1,19–3,00) | 0,007 |
| **Exposure to violence** | | | | | |
| Yes | 22,3 |  |  | REF |  |
| No | 40,4 |  |  | 1,42 (1,03–1,95) | 0,033 |
| **Resilience** | / |  |  | 1,34 (1,26–1,41) | <0,001 |
| **Literacy; finding, understanding and evaluating information** | / |  |  | 1,11 (1,01–1,22) | 0,023 |
| **Literacy; understanding and following recommendations** | / |  |  | 1,18 (1,08–1,30) | <0,001 |

Table 3: Results of regression analysis for languishing mental health.

| **Languishing mental health** | | | | | |
| --- | --- | --- | --- | --- | --- |
|  |  | **Unadjusted model** | | **Adjusted model** | |
|  | **%** | OR (95% CI) | *p* | OR (95% CI) | *p* |
| **Sex** | | | | | |
| male | 6,6 | REF |  | REF |  |
| female | 9,4 | 1,47 (1,15–1,89) | 0,002 | 1,14 (0,82–1,58) | 0,443 |
| **Age** | | | | | |
| 18 - 24 | 14,9 | REF |  | REF |  |
| 25 - 34 | 12,9 | 0,84 (0,56–1,26) | 0,404 | 2,00 (0,99–4,04) | 0,054 |
| 35 - 44 | 6,9 | 0,43 (0,28–0,66) | <0,001 | 1,27 (0,57–2,86) | 0,559 |
| 45 - 54 | 5,3 | 0,32 (0,20–0,52) | <0,001 | 1,10 (0,48–2,53) | 0,817 |
| 55 - 64 | 5,2 | 0,31 (0,19–0,50) | <0,001 | 1,22 (0,49–3,01) | 0,668 |
| 65 - 74 | 5,8 | 0,35 (0,21–0,59) | <0,001 | 1,73 (0,50–5,99) | 0,39 |
| 75 and more | 8,7 | 0,55 (0,33–0,91) | 0,020 | 2,57 (0,72–9,18) | 0,146 |
| **Education status** | | | | | |
| primary school or lower | 9,7 | REF |  | REF |  |
| secondary school | 7,8 | 0,79 (0,57–1,08) | 0,136 | 1,14 (0,73–1,78) | 0,555 |
| college or higher | 7,2 | 0,72 (0,50–1,04) | 0,083 | 1,11 (0,66–1,86) | 0,699 |
| **Employment status** | | | | | |
| employed, self-employed | 6,3 | REF |  | REF |  |
| student | 15,6 | 2,77 (1,92–4,00) | <0,001 | 2,28 (1,14–4,55) | 0,02 |
| retired | 6,1 | 0,97 (0,70–1,34) | 0,842 | 0,65 (0,27–1,52) | 0,317 |
| unemployed | 18,8 | 3,48 (2,37–5,10) | <0,001 | 2,55 (1,52–4,28) | <0,001 |
| **Marital status** | | | | | |
| married | 4,6 | REF |  | REF |  |
| civil partner | 7,6 | 1,75 (1,23–2,47) | 0,002 | 1,30 (0,81–2,08) | 0,277 |
| single | 14,9 | 3,69 (2,69–5,06) | <0,001 | 2,77 (1,73–4,43) | 2E-05 |
| widowed | 10,2 | 2,43 (1,47–4,01) | 0,001 | 1,53 (0,71–3,29) | 0,278 |
| divorced | 11,5 | 2,73 (1,45–5,14) | 0,002 | 2,60 (1,19–5,67) | 0,017 |
| **Alcohol consumption** | | | | | |
| Decreased | 6,7 | 0,83 (0,59–1,17) | 0,287 | 0,62 (0,40–0,96) | 0,032 |
| Unchanged | 7,9 | REF |  | REF |  |
| Increased | 16,7 | 2,34 (1,49–3,68) | <0,001 | 1,13 (0,63–2,00) | 0,687 |
| **COVID-19 infection** | | | | | |
| Yes | 7,4 | 1,14 (0,81–1,61) | 0,45 | 1,02 (0,67–1,54) | 0,941 |
| No | 8,4 | REF |  | REF |  |
| **Family relations** | | | | | |
| Worsened | 17,9 | 2,97 (2,25–3,91) | <0,001 | 1,57 (1,06–2,31) | 0,023 |
| Unchanged | 6,9 | REF |  | REF |  |
| Improved | 4,4 | 0,63 (0,43–0,93) | 0,021 | 0,91 (0,57–1,44) | 0,673 |
| **Financial security** | | | | | |
| Worsened | 12,6 | 2,38 (1,84–3,08) | <0,001 | 1,10 (0,78–1,56) | 0,593 |
| Unchanged | 5,7 | REF |  | REF |  |
| Improved | 5,7 | 1,01 (0,60–1,70) | 0,955 | 1,21 (0,65–2,28) | 0,547 |
| **Social interactions** | | | | | |
| Worsened | 8,9 | 1,48 (1,07–2,03) | 0,016 | 0,76 (0,51–1,15) | 0,2 |
| Unchanged | 6,2 | REF |  | REF |  |
| Improved | 4,3 | 0,64 (0,27–1,55) | 0,325 | 0,24 (0,07–0,85) | 0,027 |
| **Physical activity** | | | | | |
| Worsened | 12 | 2,45 (1,85–3,24) | <0,001 | 1,14 (0,77–1,69) | 0,509 |
| Unchanged | 5,3 | REF |  | REF |  |
| Improved | 4,2 | 0,79 (0,49–1,28) | 0,341 | 0,67 (0,36–1,26) | 0,214 |
| **Diet** | | | | | |
| Worsened | 19 | 3,75 (2,87–4,90) | <0,001 | 1,87 (1,27–2,74) | 0,002 |
| Unchanged | 5,9 | REF |  | REF |  |
| Improved | 5,2 | 0,86 (0,57–1,30) | 0,477 | 0,72 (0,40–1,31) | 0,286 |
| **Sleep** | | | | | |
| Worsened | 16,1 | 3,77 (2,90–4,92) | <0,001 | 1,40 (0,97–2,02) | 0,075 |
| Unchanged | 4,8 | REF |  | REF |  |
| Improved | 5,8 | 1,23 (0,77–1,95) | 0,384 | 1,20 (0,64–2,22) | 0,57 |
| **Health status** | | | | | |
| Worsened | 16,5 | 3,09 (2,39–4,00) | <0,001 | 1,50 (1,06–2,14) | 0,024 |
| Unchanged | 6,0 | REF |  | REF |  |
| Improved | 5,3 | 0,85 (0,36–1,99) | 0,700 | 0,76 (0,21–2,70) | 0,668 |
| **Pre-existing chronic conditions** | | | | | |
| Yes | 8,1 | 1,01 (0,78–1,32) | 0,918 | 1,01 (0,70–1,46) | 0,961 |
| No | 7,9 | REF |  | REF |  |
| **Pre-existing mental disorders** | | | | | |
| Yes | 22,4 | 3,70 (2,57–5,34) | <0,001 | 1,60 (0,98–2,62) | 0,062 |
| No | 7,2 | REF |  | REF |  |
| **Exposure to violence** | | | | | |
| Yes | 17,6 | 2,90 (2,14–3,93) | <0,001 | 1,33 (0,88–2,01) | 0,172 |
| No | 6,8 | REF |  | REF |  |
| **Resilience** | / | 0,64 (0,59–0,69) | <0,001 | 0,72 (0,65–0,80) | <0,001 |
| **Literacy; finding, understanding and evaluating information** | / | 0,70 (0,64–0,77) | <0,001 | 0,78 (0,68–0,90) | 8E-04 |
| **Literacy; understanding and following recommendations** | / | 0,79 (0,72–0,86) | <0,001 | 1,05 (0,91–1,21) | 0,511 |

Table 4: Multinomial regression models for languishing mental health.

| **Languishing mental health** | | | | | |
| --- | --- | --- | --- | --- | --- |
|  |  | **Unadjusted model** | | **Adjusted model** | |
|  | **%** | OR (95% CI) | *p* | OR (95% CI) | *p* |
| **Sex** | | | | | |
| male | 6,6 | REF |  | REF |  |
| female | 9,4 | 1,47 (1,1–1,97) | 0,010 | 1,14 (0,82–1,58) | 0,443 |
| **Age** | | | | | |
| 18 - 24 | 14,9 | REF |  | REF |  |
| 25 - 34 | 12,9 | 1,56 (0,84–2,91) | 0,163 | 2,00 (0,99–4,04) | 0,054 |
| 35 - 44 | 6,9 | 0,88 (0,43–1,79) | 0,720 | 1,27 (0,57–2,86) | 0,559 |
| 45 - 54 | 5,3 | 0,82 (0,39–1,71) | 0,592 | 1,10 (0,48–2,53) | 0,817 |
| 55 - 64 | 5,2 | 0,98 (0,44–2,17) | 0,956 | 1,22 (0,49–3,01) | 0,668 |
| 65 - 74 | 5,8 | 1,23 (0,4–3,75) | 0,721 | 1,73 (0,50–5,99) | 0,39 |
| 75 and more | 8,7 | 2,03 (0,64–6,43) | 0,227 | 2,57 (0,72–9,18) | 0,146 |
| **Education status** | | | | | |
| primary school or lower | 9,7 | REF |  | REF |  |
| secondary school | 7,8 | 1,01 (0,68–1,51) | 0,961 | 1,14 (0,73–1,78) | 0,555 |
| college or higher | 7,2 | 0,87 (0,54–1,39) | 0,554 | 1,11 (0,66–1,86) | 0,699 |
| **Employment status** | | | | | |
| employed, self-employed | 6,3 | REF |  | REF |  |
| student | 15,6 | 1,64 (0,88–3,05) | 0,118 | 2,28 (1,14–4,55) | 0,02 |
| retired | 6,1 | 0,71 (0,32–1,58) | 0,400 | 0,65 (0,27–1,52) | 0,317 |
| unemployed | 18,8 | 2,6 (1,67–4,05) | <0,001 | 2,55 (1,52–4,28) | <0,001 |
| **Marital status** | | | | | |
| married | 4,6 | REF |  | REF |  |
| civil partner | 7,6 | 1,56 (1,01–2,42) | 0,044 | 1,30 (0,81–2,08) | 0,277 |
| single | 14,9 | 3,1 (2–4,82) | <0,001 | 2,77 (1,73–4,43) | <0,001 |
| widowed | 10,2 | 1,77 (0,85–3,7) | 0,13 | 1,53 (0,71–3,29) | 0,278 |
| divorced | 11,5 | 3,03 (1,49–6,16) | 0,002 | 2,60 (1,19–5,67) | 0,017 |
| **Alcohol consumption** | | | | | |
| Decreased | 6,7 |  |  | 0,62 (0,40–0,96) | 0,032 |
| Unchanged | 7,9 |  |  | REF |  |
| Increased | 16,7 |  |  | 1,13 (0,63–2,00) | 0,687 |
| **COVID-19 infection** | | | | | |
| Yes | 7,4 |  |  | 1,02 (0,67–1,54) | 0,941 |
| No | 8,4 |  |  | REF |  |
| **Family relations** | | | | | |
| Worsened | 17,9 |  |  | 1,57 (1,06–2,31) | 0,023 |
| Unchanged | 6,9 |  |  | REF |  |
| Improved | 4,4 |  |  | 0,91 (0,57–1,44) | 0,673 |
| **Financial security** | | | | | |
| Worsened | 12,6 |  |  | 1,10 (0,78–1,56) | 0,593 |
| Unchanged | 5,7 |  |  | REF |  |
| Improved | 5,7 |  |  | 1,21 (0,65–2,28) | 0,547 |
| **Social interactions** | | | | | |
| Worsened | 8,9 |  |  | 0,76 (0,51–1,15) | 0,2 |
| Unchanged | 6,2 |  |  | REF |  |
| Improved | 4,3 |  |  | 0,24 (0,07–0,85) | 0,027 |
| **Physical activity** | | | | | |
| Worsened | 12 |  |  | 1,14 (0,77–1,69) | 0,509 |
| Unchanged | 5,3 |  |  | REF |  |
| Improved | 4,2 |  |  | 0,67 (0,36–1,26) | 0,214 |
| **Diet** | | | | | |
| Worsened | 19 |  |  | 1,87 (1,27–2,74) | 0,002 |
| Unchanged | 5,9 |  |  | REF |  |
| Improved | 5,2 |  |  | 0,72 (0,40–1,31) | 0,286 |
| **Sleep** | | | | | |
| Worsened | 16,1 |  |  | 1,40 (0,97–2,02) | 0,075 |
| Unchanged | 4,8 |  |  | REF |  |
| Improved | 5,8 |  |  | 1,20 (0,64–2,22) | 0,57 |
| **Health status** | | | | | |
| Worsened | 16,5 |  |  | 1,50 (1,06–2,14) | 0,024 |
| Unchanged | 6,0 |  |  | REF |  |
| Improved | 5,3 |  |  | 0,76 (0,21–2,70) | 0,668 |
| **Pre-existing chronic conditions** | | | | | |
| Yes | 8,1 |  |  | 1,01 (0,70–1,46) | 0,961 |
| No | 7,9 |  |  | REF |  |
| **Pre-existing mental disorders** | | | | | |
| Yes | 22,4 |  |  | 1,60 (0,98–2,62) | 0,062 |
| No | 7,2 |  |  | REF |  |
| **Exposure to violence** | | | | | |
| Yes | 17,6 |  |  | 1,33 (0,88–2,01) | 0,172 |
| No | 6,8 |  |  | REF |  |
| **Resilience** | / |  |  | 0,72 (0,65–0,80) | <0,001 |
| **Literacy; finding, understanding and evaluating information** | / |  |  | 0,78 (0,68–0,90) | 8E-04 |
| **Literacy; understanding and following recommendations** | / |  |  | 1,05 (0,91–1,21) | 0,511 |
