# Supplementary material for: Positive mental health in Slovenia before and during the COVID-19 pandemic
Source: Front Public Health. 2022 Oct 14;10:963545. doi: 10.3389/fpubh.2022.963545 (PMC9614317; doi:10.3389/fpubh.2022.963545)
Supplement: Supplementary file 2 [file Table_2.DOCX]

Supplementary Material

# Appendix 1: Sociodemographic characteristics of study participants

Table 1: Table on socio-demographic characteristics of EHIS and SI-PANDA samples

|  | **EHIS 2019** | | | **SI-PANDA 2021** | | |
| --- | --- | --- | --- | --- | --- | --- |
|  | Unweighted | | Weighted | Unweighted | | Weighted |
|  | N | % | % | N | % | % |
| **Sex** | | | | | | |
| male | 4059 | 44.9 | 49.4 | 1617 | 46.7 | 51.0 |
| female | 4988 | 55.1 | 50.6 | 1845 | 53.3 | 49.0 |
| **Age (years)** |  |  |  |  |  |  |
| 18 - 24 | 785 | 8.7 | 8.4 | 245 | 7.1 | 8.7 |
| 25 - 34 | 1089 | 12.0 | 15.1 | 399 | 11.6 | 15.7 |
| 35 - 44 | 1545 | 17.1 | 18.6 | 520 | 14.9 | 19.2 |
| 45 - 54 | 1614 | 17.8 | 17.7 | 570 | 16.5 | 18.2 |
| 55 - 64 | 1679 | 18.5 | 17.4 | 748 | 21.6 | 17.3 |
| 65 - 74 | 1344 | 14.9 | 12.9 | 592 | 17.1 | 12.0 |
| 75 and more | 991 | 11.0 | 9.9 | 388 | 11.2 | 8.9 |
| **Marital status** | | | | | | |
| married | 4672 | 51.9 | 50.2 | 1732 | 50.3 | 45.3 |
| civil partner | 1253 | 13.9 | 15.0 | 764 | 22.0 | 24.7 |
| single | 1755 | 19.5 | 21.0 | 573 | 16.7 | 20.7 |
| widowed | 812 | 9.0 | 8.5 | 256 | 7.3 | 6.1 |
| divorced | 516 | 5.7 | 5.3 | 126 | 3.7 | 3.2 |
| **Education status** | | | | | | |
| primary education or lower | 1264 | 14.0 | 19.5 | 419 | 12.1 | 17.8 |
| secondary education | 4963 | 54.8 | 54.7 | 1742 | 50.3 | 55.0 |
| college or higher | 2820 | 31.2 | 25.8 | 1301 | 37.6 | 27.2 |
| **Employment status** | | | | | | |
| employed, self-employed | 4609 | 51.2 | 53.8 | 1763 | 51.0 | 56.7 |
| student | 576 | 6.4 | 6.1 | 239 | 6.9 | 8.3 |
| retired | 3045 | 33.8 | 30.4 | 1201 | 34.6 | 26.2 |
| unemployed | 507 | 5.6 | 6.4 | 178 | 5.2 | 6.4 |
| other | 265 | 3.0 | 3.3 | 77 | 2.3 | 2.4 |

# Appendix 2: Forest plot of the Adjusted model 2

Figure 1: Forest plot of the adjusted logistic regression evaluating odds for flourishing mental health

Figure 2: Forest plot of the adjusted logistic regression evaluating odds for languishing mental health
